# Supplementary material for: Development of an AI-Empowered Novel Digital Monitoring System for Inhalation Flow Profiles
Source: Sensors (Basel). 2025 Jul 15;25(14):4402. doi: 10.3390/s25144402 (PMC12299986; doi:10.3390/s25144402)
Supplement: Supplementary file 1 [file sensors-25-04402-s001.zip › sensors-3681312-supplementary.pdf]

sensors MDPI

Development of an AI-Empowered Novel Digital Monitoring System for Inhalation Flow Profiles

Frameworks of Voting Ensembles, Stacking Ensembles, and Blending Ensembles

Voting is one of the fundamental heterogeneous methods in machine learning. In the training stage (Level 0 as illustrated in Figure S1), multiple base learners are trained independently on the same dataset. Following this, the final prediction in output stage (Level 1) is commonly obtained by calculating the average of the predictions generated by each base model in training stage (Level 0). This averaging approach helps to mitigate individual model errors as well as enhance the robustness and generalizability of the ensemble's prediction.

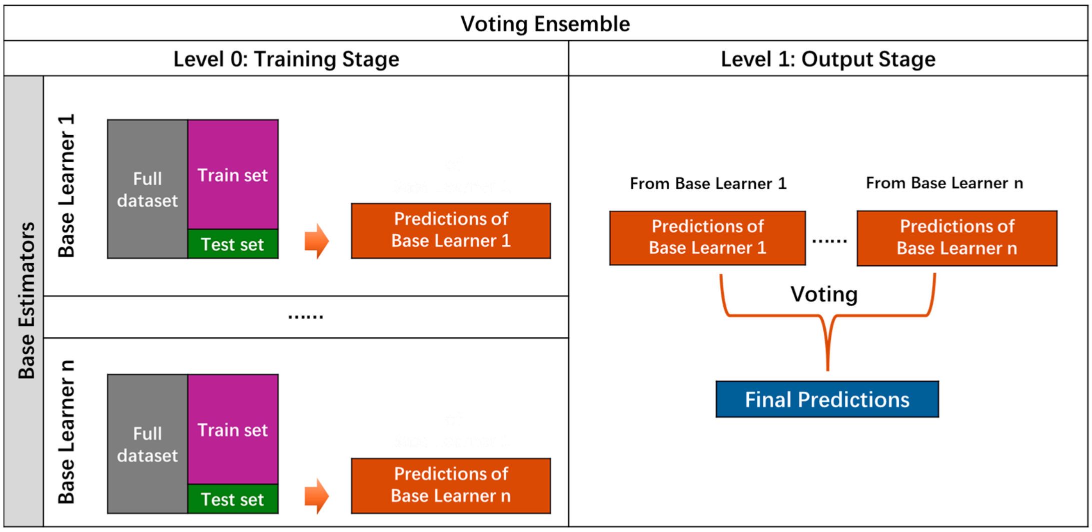

.

Figure S1. Illustration of the voting ensemble framework. Level 0 involves independent training on the same dataset using multiple base learners. Level 1 then calculates the average of the predictions obtained from Level 0 to obtain the final predictions.

The framework for the stacking ensemble strategy is illustrated in Figure S2. Stacking is an advanced ensemble technique by which multiple base models are trained (Level 0), and their predictions are then integrated through a meta-model (Level 1) to enhance predictive accuracy and generalizability. In this study, a 5-fold cross-validation strategy was employed to train the meta-model, therefore improving its robustness and mitigating overfitting.

 https://doi.org/10.3390/xxxxx

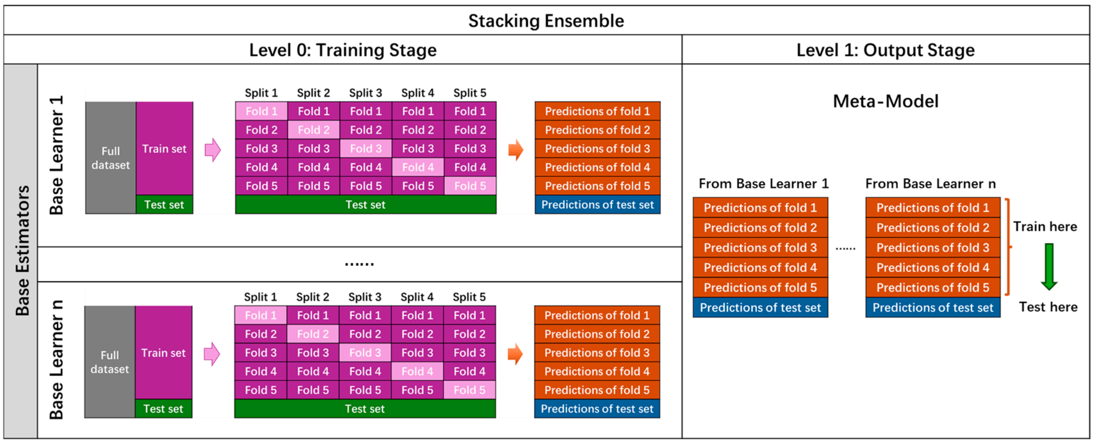

.

Figure S2. Illustration of stacking ensemble framework. Level 0 involves the training of base learners. Level 1 deploys a meta-model, which is trained on the predictions originating from the trained base learners in Level 0.

Blending ensemble shares similarities with stacking, as both methods use base learners to generate predictions, which are then treated as new features for a meta-model that makes the final prediction. However, blending differs by incorporating a holdout set to train the meta-model on these new features. The blending framework is illustrated in Figure S3.

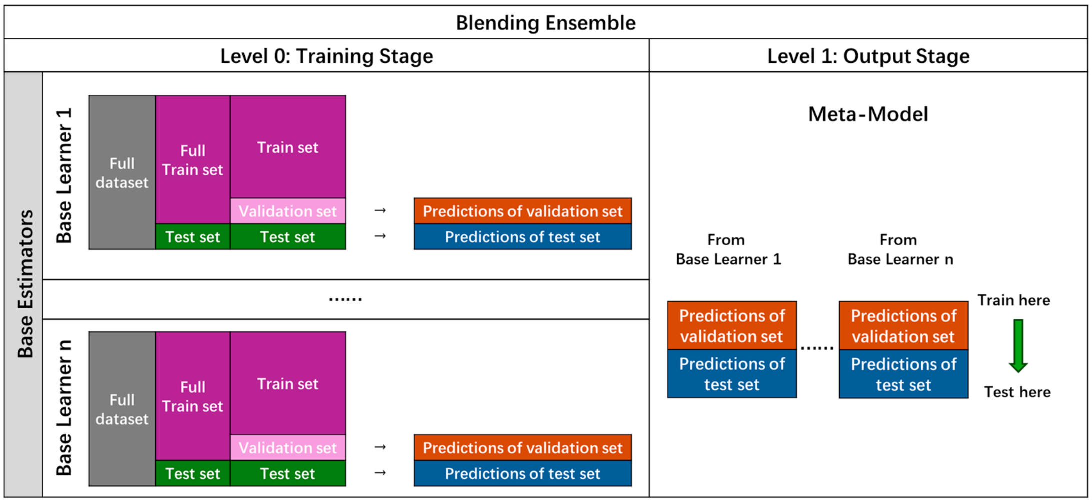

.

Figure S3. Illustration of blending ensemble framework. In Level 0, base learners are trained using a straightforward holdout set approach to generate initial predictions. In Level 1, a meta-model is trained on the predictions generated, leveraging the outputs of the base learners to refine and enhance the overall predictive performance.

Table S1. The training time for each algorithm.

ML Algorithms	Training Time (Grid Search Cross-Validation)
Decision Tree	7min 56s
Random Forest (RF)	3h 3min 34s
Extra Trees Regressor (ETR)	1h 21min 8s

Support Vector Machine (SVM)	1h 38min 32s
Gaussian Progress Regressor	2h 32min 36s
AdaBoost	15min 2s
XGBoost	1h 50min 55s
GradientBoosting	2h 16min 47s

Table S2. Values of fine-tuned optimal hyperparameters for the base learner models.

ML Algorithms	Values of Hyperparameters
Decision Tree	max_depth = 10, min_samples_leaf = 16
Random Forest (RF)	max_depth = 20, max_features = 2, min_samples_leaf = 2, min_samples_split = 2, n_estimators = 200
Extra Trees Regressor (ETR)	max_depth = 20, max_features = 2, min_samples_leaf = 2, min_samples_split = 2, n_estimators = 200
Support Vector Machine (SVM)	C = 20, epsilon = 0.5, gamma = 1, kernel = 'rbf'
Gaussian Progress Regressor	alpha = 10, kernel= 1**2 * RBF(length_scale=1)
AdaBoost	learning_rate = 0.1, loss = 'linear', n_estimators = 200
XGBoost	colsample_bytree = 0.7, learning_rate = 0.1, max_depth = 7, min_child_weight = 2, n_estimators = 150, subsample = 0.7
GradientBoosting	learning_rate = 0.01, max_depth = 4, min_samples_leaf = 8, min_samples_split = 2, n_estimators = 300

Table S3. The structures of the heterogeneous ensemble frameworks.

ML Algorithms	Structure
Voting Ensemble_1	RF + AdaBoost + ETR + XGBoost
Voting Ensemble_2	RF + AdaBoost + ETR
Voting Ensemble_3	RF + ETR
Voting Ensemble_4	RF + ADA
Stacking Ensemble_1	AdaBoost + ETR + XGBoost; Meta model = RF
Stacking Ensemble_2	RF + ETR + XGBoost; Meta model = AdaBoost
Stacking Ensemble_3	RF + AdaBoost + XGBoost; Meta model = ETR
Stacking Ensemble_4	RF + AdaBoost + ETR; Meta model = XGBoost
Stacking Ensemble_5	ETR; Meta model = RF
Stacking Ensemble_6	RF; Meta model = ETR
Blending Ensemble_1	AdaBoost + ETR + XGBoost; Meta model = RF
Blending Ensemble_2	RF + ETR + XGBoost; Meta model = AdaBoost
Blending Ensemble_3	AdaBoost + ETR + XGBoost; Meta model = ETR
Blending Ensemble_4	RF+ AdaBoost +ETR; Meta model = XGBoost

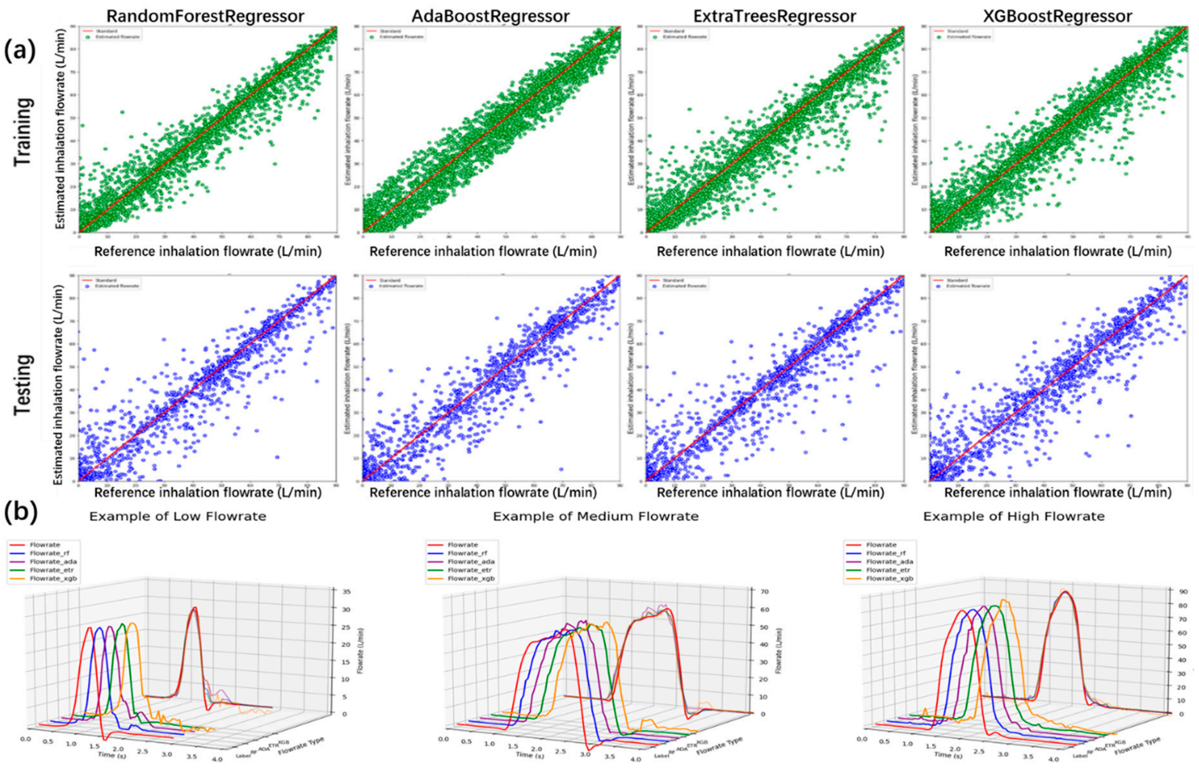

Figure S4. (a) Scatter plots of "predicted flowrate values" versus "referenced flowrate values" for top four base learner models on both the training and testing subsets; (b) Representative inhalation flow profiles for the top four base learner models in low, medium, and high flowrate ranges.

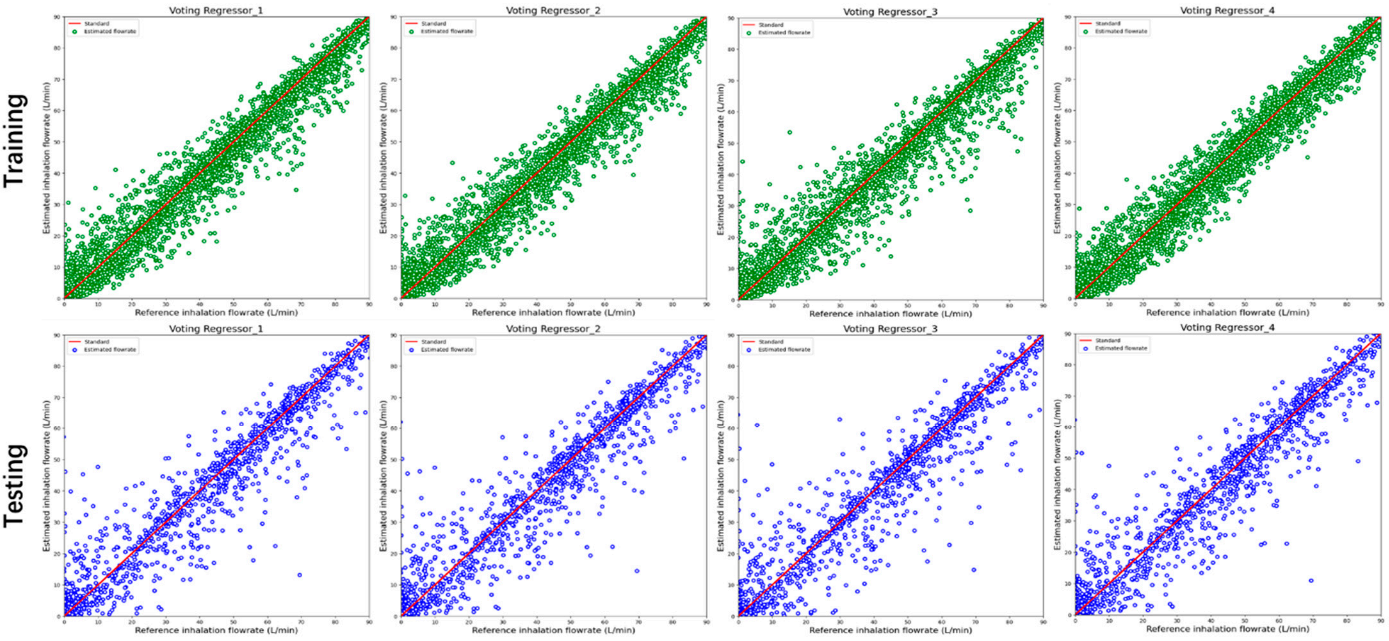

Figure S5. Scatter plots of voting ensemble models 'predicted Flowrate values' vs. 'reference flowrate values' in the training and testing subsets.

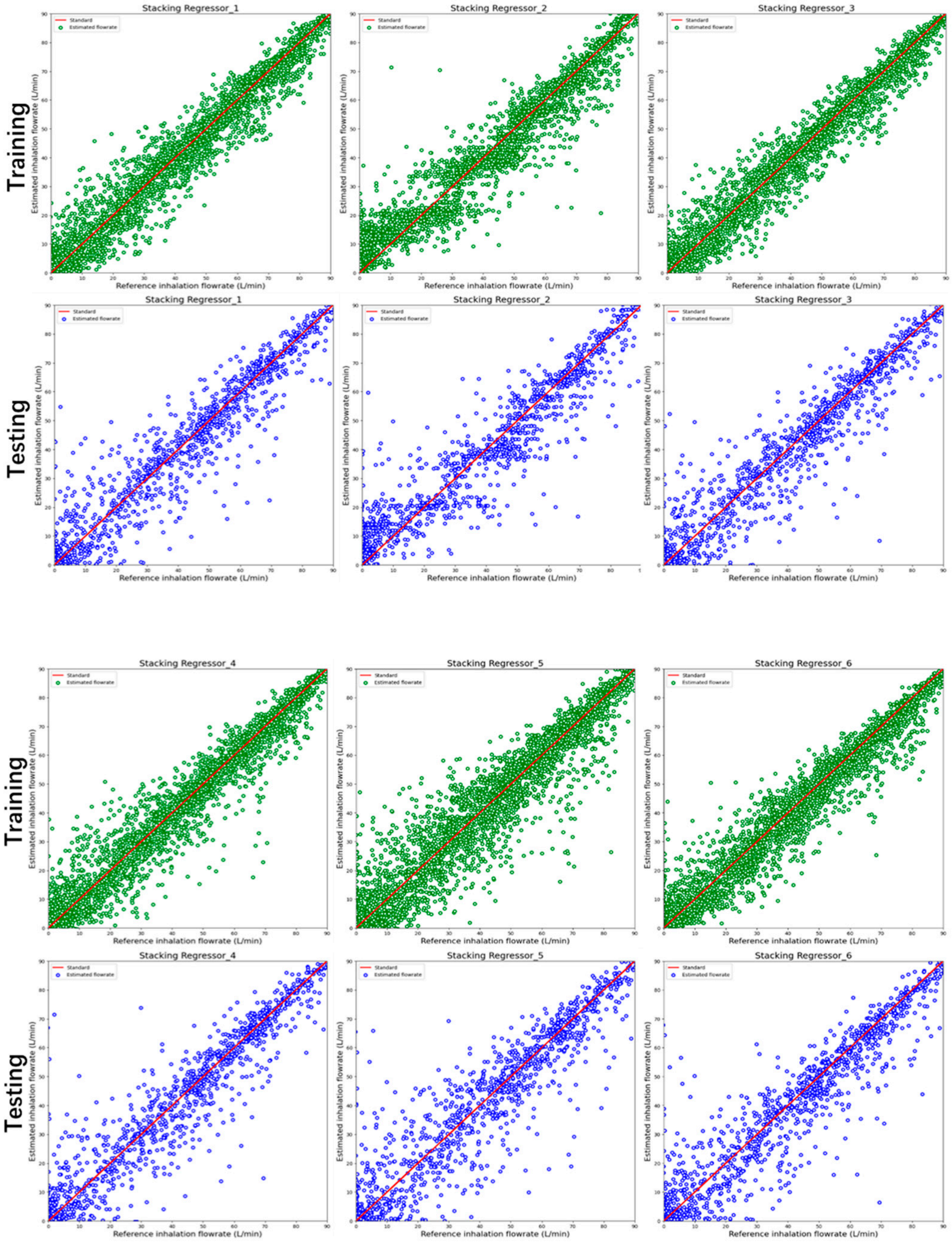

Figure S6. Scatter plots of stacking ensemble models 'predicted Flowrate values' vs. 'reference flowrate values' in the training and testing subsets.

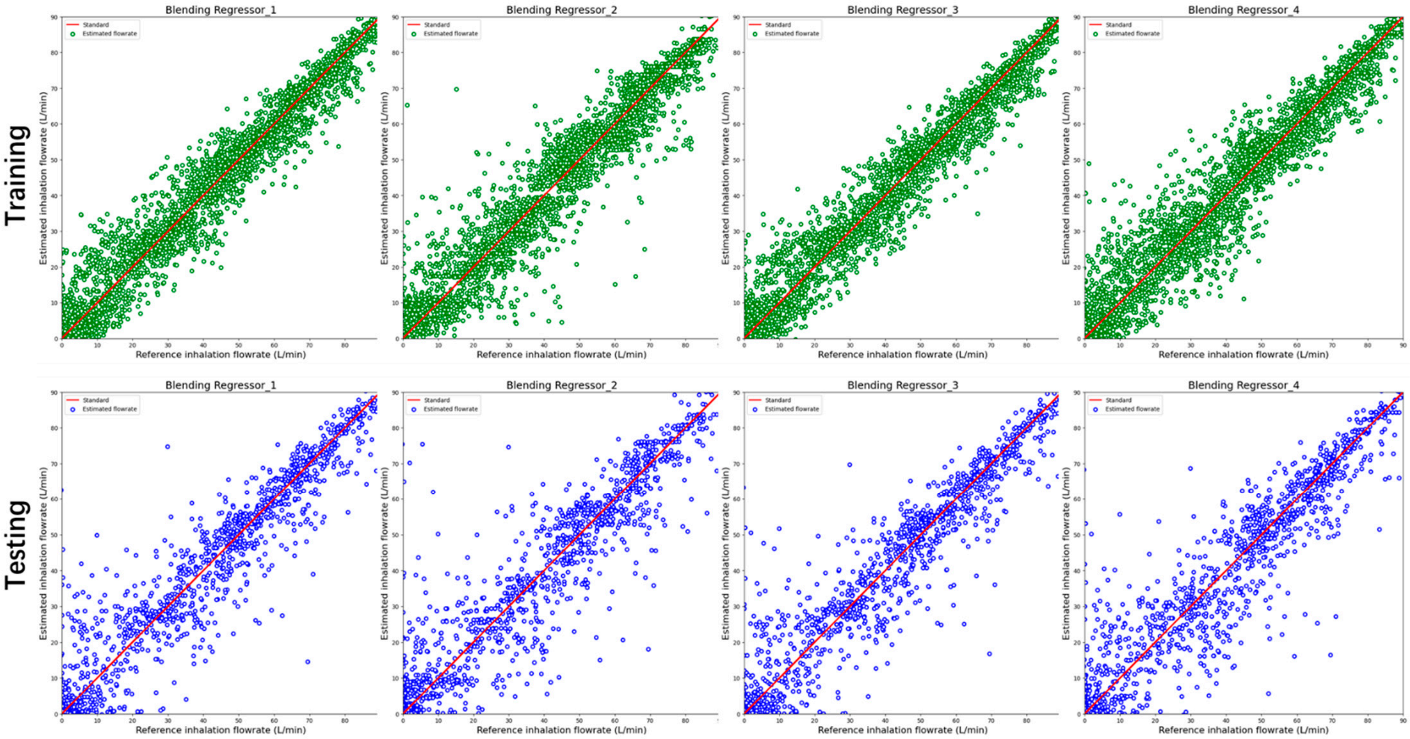

Figure S7. Scatter plots of blending ensemble models 'predicted Flowrate values' vs. 'reference flowrate values' in the training and testing subsets.

Table S4. Case examples of estimated PIFR and IC using the optimal base learner, voting ensemble, stacking ensemble, and blending ensemble models.

ML Algorithms	Predicted PIFR (L/min)	Relative Error (%)	Predicted IC (L)	Relative Error (%)
**Best Single Base Learner (**Random Forest**)**	80.122	1.500	2.150	1.896
**Best Voting Ensemble (**Voting Ensemble 1**)**	80.693	0.798	2.148	1.801
**Best Stacking Ensemble (**Stacking Ensemble 3**)**	83.136	2.206	2.139	1.374
**Best Blending Ensemble (**Blending Ensemble 3**)**	81.071	0.333	2.155	2.133

Clinical Relevance Analysis

To preliminarily explore the clinical relevance of the proposed system, we performed a binary classification analysis based on a commonly used clinical threshold of PIFR ≥ 50 L/min for Breezhaler® [40]. The best-performing model (Random Forest) achieved an accuracy of 98.75%, sensitivity of 100.00%, and a Cohen's kappa of 0.97, indicating strong agreement with the ground truth and excellent ability to detect insufficient inhalation effort. While encouraging, these findings remain preliminary due to the absence of patient data, underscoring the need for further validation in diverse clinical populations to confirm the system's robustness and real-world applicability.

Table S5. Performance of the models in classifying sufficient inhalation effort based on a clinical threshold of PIFR ≥ 50 L/min. Metrics include classification accuracy, sensitivity, specificity, Cohen's kappa, and confusion matrix elements (true positives [TP], false positives [FP], true negatives [TN], and false negatives [FN]).

ML Models	Accuracy (%)	Sensitivity (%)	Specificity (%)	Cohen's Kappa	TP	FP	TN	FN
**Best Single Base Learner**	98.75	100.00	95.12	0.97	119	2	39	0

(Random Forest)								
**Best Voting Ensemble** (Voting Ensemble 1)	97.50	98.32	95.12	0.93	117	2	39	2
**Best Stacking Ensemble** (Stacking Ensemble 3)	96.88	100.00	87.80	0.91	119	5	36	0
**Best Blending Ensemble** (Blending Ensemble 3)	95.62	100.00	82.93	0.88	119	7	34	0

References

40. Hassan, M.I.; Laz, N.I.; Madney, Y.M.; Harb, H.S.; Abdelrahim, M.E.A. Enhancing Chronic Obstructive Pulmonary Disease Management through Optimized Peak Inspiratory Flow Rate and Inhaler Strategies: Literature Review. *Bull. Pharm. Sci. Assiut Univ.* **2024**, *0*, 0–0, doi:10.21608/bfsa.2024.306558.2209.
